# Immunogenicity of Recombinant Hepatitis B Vaccine Among Routinely Vaccinated Healthy and Chronically Ill Children in Assiut, Upper Egypt

**DOI:** 10.14740/gr636e

**Published:** 2015-07-22

**Authors:** Osama M. El-Asheer, Manal M. Darwish, Madleen A. Abdou, Khaled Saad

**Affiliations:** a Department of Pediatrics, Faculty of Medicine, Assiut University, Assiut, Egypt; b Department of Public Health & Community Medicine, Faculty of Medicine, Assiut University, Assiut, Egypt; c Department of Clinical Pathology, Faculty of Medicine, Assiut University, Assiut, Egypt

**Keywords:** Immunogenicity, Hepatitis B vaccine, Children

## Abstract

**Background:**

Egypt is considered a region of the intermediate prevalence of hepatitis B virus (HBV) infection (4.5%). Seroprotection is assured when hepatitis B surface antibody (HBsAb) levels are ≥ 10 mIU/mL. Our study aimed to evaluate and compare the long-term immunogenicity and efficacy of the recombinant hepatitis B (HB) vaccine.

**Methods:**

A cross-sectional study was done for children aged from 9 months to 15 years, receiving health care at Assiut University Children’s Hospital, Assiut, Egypt in 3 months. HBsAb was quantitatively determined by enzyme-linked immune sorbent assay (ELISA).

**Results:**

Seroprotection in infants less than 1 year was 89.7% with 55.2% having titer > 100 mIU/mL and this percent dropped to 64.4% after the first year of age with only 29% having titer > 100 mIU/mL. The overall protection percentage was 32.5% (> 100 mIU/mL), 34.7% of children showed levels between 10 and 100 mIU/mL, while 32.8% were less than 10 mIU/mL. Patients with diabetes mellitus were found to have the lowest seroprotective levels (83.3% were not protected). Non-protective levels were also detected in patients with malnutrition (55.6%), congenital heart diseases (43.2%) and chronic liver diseases (57.1%).

**Conclusion:**

Our study shows failure to achieve satisfactory seroprotective levels for hepatitis B vaccine in both healthy and diseased children who adopted vaccination schedule in Upper Egypt. Booster dose in the second year of life is recommended for all children, particularly for those with diabetes millets, congenital heart disease and malnutrition.

## Introduction

Hepatitis B virus (HBV) infection is the most widespread chronic infectious disease worldwide. Nearly one-third of the world’s population has serological evidence of past or present infection with HBV. An estimated 400 million persons worldwide are chronically infected with HBV. The global prevalence of HBV varies widely from low (< 2% as in Western Europe, North America and Japan) to high (> 8% as in Africa, Southeast Asia and China). HBV infection is a major health problem in Egypt. Egypt is considered to be a region of intermediate prevalence for HBV infection with a reported figure of 4.5%. Nearly 2 - 3 million Egyptians are chronic carriers of HBV [1, 2].

Infection of infants and young children with HBV represents an important health hazard, since the younger the age at which the infection is acquired, the greater the predisposition to the carrier state, chronic liver disease and subsequent development of cirrhosis and hepatocellular carcinoma [1, 2].

Safe and effective vaccines against hepatitis B (HB) infection have been available since 1982. The World Health Organization (WHO) recommended universal vaccination against HB; this recommendation had been applied in Egypt. A compulsory vaccination program against HBV among infants was started in Egypt in 1992 using a yeast recombinant DNA vaccine (10 µg) and with a schedule of 2, 4 and 6 months in age [1, 3, 4].

The duration of protection after HB vaccination of infants is unknown, although many studies have found that after neonatal immunization with HB vaccine, a large proportion of the children, especially adolescents, exhibited waning immunity. Such decreased protection poses the risk of development infection [3-5].

Although advantageous for practical causes to incorporate HB vaccination into the schedule of the routine childhood immunization program, many authors have shown that short intervals of 1 or 2 months between the second and third doses of HB vaccine are accompanied by significantly lower levels of antibodies to HB surface antigen (anti-HBs) when compared to longer intervals of 4 months or more [2, 5, 6].

The aim of our study is to evaluate and compare the long-term immunogenicity and efficacy of the recombinant HB vaccine administered to infants, according to the Egypt’s National Immunization Program in both normal and chronically diseased children.

## Patients and Methods

### Study population

This was a cross-sectional study. The participants were recruited from infants and children who were receiving health care at Assiut University Children’s Hospital, Assiut, Egypt. The study was approved by the Researches and Ethical Review Committee, Faculty of Medicine, Assiut University. The date and dose intervals of HB vaccine were confirmed by checking the vaccination record written on the birth certificate of each child. The purpose of the study was carefully explained to the child’s parents or guardians and informed consent to participate in the study was obtained before blood sampling.

A total of 262 children met the eligibility criteria which include: 1) age ranged from 9 months to 15 years; 2) born to hepatitis B surface antigen (HBsAg) negative mothers; 3) no history of prematurity; 4) had completed primary HB vaccination program according to the Egypt’s National Immunization Program. A questionnaire was designed and administered to the parents or caretakers of the children to collect the basic demographic data (age, sex, etc.).

### Blood sampling and serological test

Venous blood samples were collected from participants under standardized conditions. Samples were centrifuged (3,000 g for 10 min) and serum samples were stored in aliquots at -20 °C until analysis. Hepatitis B surface antibody (HBsAb) was quantitatively determined by enzyme-linked immune sorbent assay (ELISA) kit code number DEH02 from DIAKEY-Korea, according to manufacturer’s instructions.

The vaccine-induced antibody response was taken to be the anti-HBs level at 1 month following the third vaccine dose. Children with non-measurable (0.0) anti-HBs titers were considered seronegative (non-responders) and those with anti-HBs levels < 10 mIU/mL were considered to have an inadequate response. These two groups were not seroprotected. Those with anti-HBs levels ≥ 10 mIU/mL were considered to be seroprotected taking into consideration that those with anti-HBs levels between 10 mIU/mL and 100 mIU/mL were rated as having a low immune response needing a booster dose to have an adequate immune response and those with anti-HBs levels > 100 mIU/mL were rated as having a good immune response to the HB vaccine [2, 7, 8].

### Statistical analysis

Collected data were analyzed using SPSS, version 16. Descriptive analysis (mean and SD) was performed. Appropriate tests of significance were used to show statistical differences (*t*-test, Chi-square, one-way ANOVA, etc.). To detect the relation between age and antibody titer, a correlation test was used. P-value was considered significant if < 0.05.

## Results

**Figure 1:**
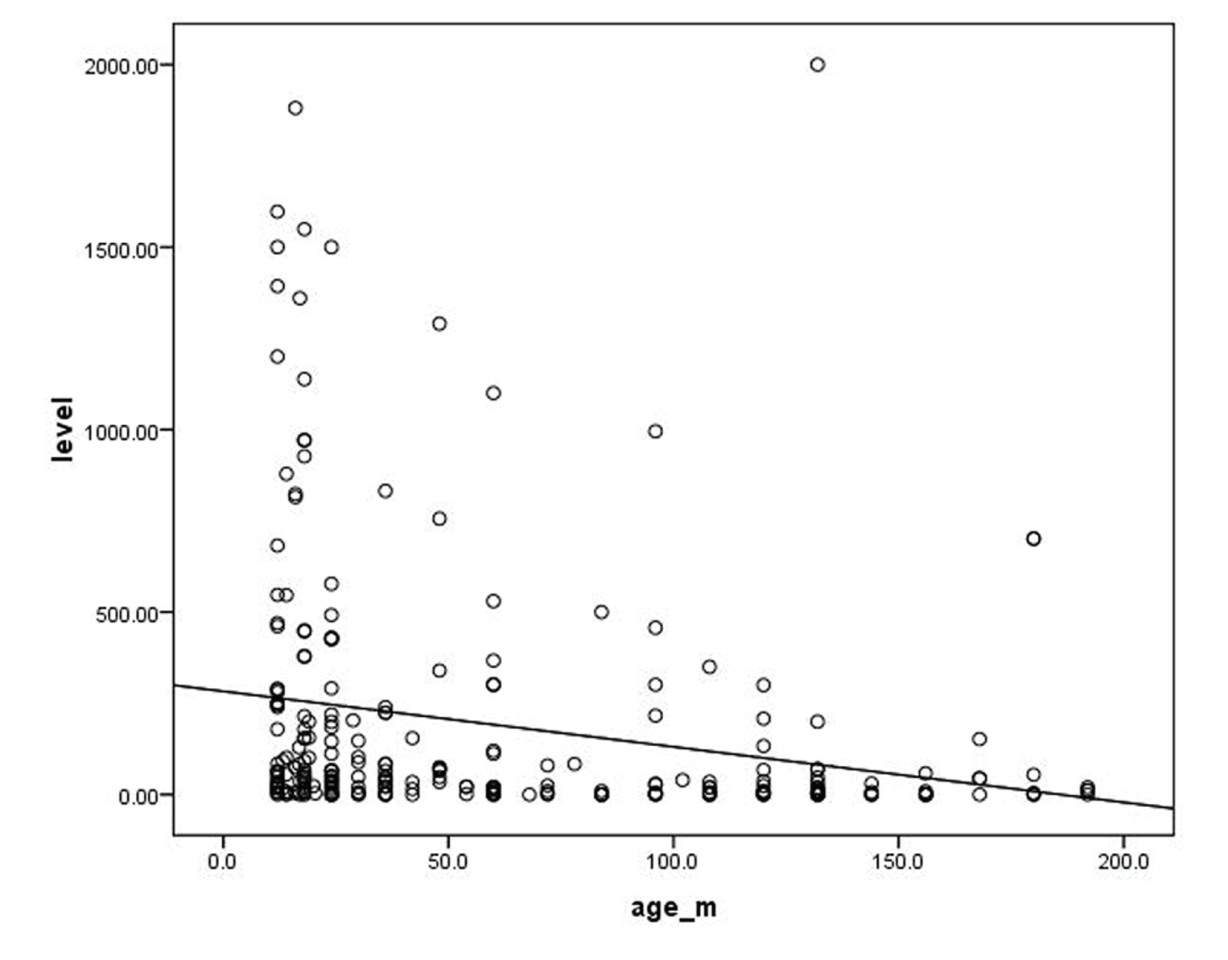
Correlation between age (in months) of the participating children and their Ab titer (r = -0.2; P = 0.0001).

Our study included 262 children (129 female, 49%) aged from 9 months to 15 years. Eleven point one percent were under 1 year and 88.9% were from 1 to 15 years. All children were negative for HBsAg and had no history or clinical evidence of HBV infection. About half of the children in our study were clinically normal. Among the diseased children, congenital heart diseases were the most common (14.1%), followed by renal failure (11.4%), while chronic liver diseases came at the bottom of the list (2.7%) (Table 1). Table 2 shows the distribution of seroprevalence of HBsAb titer by health status. Seroprevalence of HBsAb titer was found to be below 100 mIU/mL in malnutrition, congenital heart disease, chronic liver disease and diabetes mellitus (in order from highest to lowest). More than half of our children aged less than 1 year had antibody (Ab) titer of ≥ 100 mIU/mL compared to only 29.6% of those aged more than 1 year with a statistical significant difference (Table 3). No significant difference was detected between males and females in the immunological response to HBV vaccine. There was a significant difference in the Ab titer between children aged less than 5 years and those aged from 5 to less than10 years (P = 0.02). This difference became significantly higher when compared with those aged more than10 years (P = 0.001). However, there was no statistical significant difference in the Ab titer between the older two groups (P = 0.5) (Table 4). There was a highly significant difference in the Ab titer between normal children compared to diabetic children followed by children with congenital heart diseases than those with malnutrition (P value: 0.0001, 0.017 and 0.021 respectively) (Table 5). Figure 1 shows significant negative correlation between the age of the participating children and their Ab titer (r = -0.2; P = 0.0001).

**Table 1:** Distribution of Studied Children by Health Status

	Frequency	Percent (%)
Normal	139	53
Renal failure	30	11.4
Diabetes mellitus	12	4.6
Congenital heart diseases	37	14.1
Malnutrition	18	6.9
Chronic liver diseases	7	2.7
Chronic hemolytic anemias	19	7.3
Total	262	100

**Table 2:** Distribution of Seroprevalence of HBsAb Titer by Health Status

	Mean (mIU/mL)	SD
Normal	251.8	386.5
Renal failure	221.0	409.7
Diabetes mellitus	10.3	19.3
Congenital heart disease	53.3	85.6
Malnutrition	60.2	90.7
Chronic liver disease	46.1	81.6
Chronic hemolytic anemias	195.0	470.8
Total	186.4	350.2

**Table 3:** Distribution of Antibody Titer by Age

Age	Antibody titer (mIU/mL)			Total	P-value*
	< 10	10 - < 100	≥ 100		
One year or less (%)	3(10.3)	10 (34.5)	16 (55.2)	29 (100)	0.05*
More than one year (%)	83 (35.6)	81 (34.8)	69 (29.6)	233 (100)	
Total (%)	86 (32.8)	91(34.7)	85 (32.5)	262 (100)	

*Significant.

**Table 4:** Comparing 5-Year Age Groups Regarding Antibody Titer

	Mean difference	95% CI	P-value
Children < 5 years versus			
≥ 5 years to < 10 years	128.98	18.56 - 239.39	0.022*
≥ 10 years	169.45	67.92 - 270.97	0.001*
Children ≥ 5 years to < 10 years versus ≥ 10 years	40.47	-87.48-168.42	0.534

*Significant.

**Table 5:** Comparing Antibody Titer Between Normal Children and Others With Different Diseases

	Antibody titer (mIU/mL)			Total, N (%)	P-value
	< 10, N (%)	10 - < 100, N (%)	≥ 100, N (%)		
Normal	34 (24.5)	46 (33.1)	59 (42.4)	139 (100)	
Renal failure	8 (26.7)	14 (46.7)	8 (26.7)	30 (100)	0.237
Diabetes mellitus	10 (83.3)	2 (16.7)	0 (0.0)	12 (100)	0.0001*
Congenital heart disease	16 (43.2)	14 (37.8)	7 (18.9)	37 (100)	0.017*
Malnutrition	10 (55.6)	3 (16.7)	5 (27.8)	18 (100)	0.021*
Chronic liver disease	4 (57.1)	2 (28.6)	1 (14.3)	7 (100)	0.031*
Chronic hemolytic anemia	4 (21.1)	10 (52.6)	5 (26.3)	19 (100)	0.228

*Significant.

## Discussion

Evaluation of the long-term immunogenicity and the duration of protection afforded by the recombinant HB vaccine are limited and incomprehensible. In addition, studies have not clearly established the need as booster doses have a better immunogenic response. Both host and immunization factors affect the immunogenic response to HB vaccine and consequently can influence the duration of immunity [2, 8, 9]. Age, weight, immune competence and genetics are considered as important host factors. Vaccine-related factors such as storage, transportation, cold chain, dose, site of immunization and vaccination schedule are considered as very important factors in immunogenicity of the vaccine [7-11]. Since the introduction of recombinant HB vaccine in 1980s, multiple studies were done to determine the antibody seroprotective levels (immune threshold) that varied among studies and they concluded that anti-HBs levels < 10 mIU/mL were considered to have inadequate immune response and need to be revaccinated, those with levels between 10 and 100 mIU/mL have low immune response and need booster dose of the vaccine and those with anti-HB levels >100 mIU/mL were rated as having good immune response [2, 7-15].

Our study is the first study in Upper Egypt to assess the long-term immunogenicity and efficacy of the recombinant HB vaccine in both normal and chronically diseased children aged 9 months to 15 years. In the present study the overall seroprotection in infants less than 1 year was 89.7% with 55.2% having titer > 100 mIU/mL and this percent dropped to 64.4% after the first year of age with only 29.6% having titer > 100 mIU/mL. The overall protection in our study was 32.5% (> 100 IU/mL), 34.7% show levels between 10 and 100 mIU/mL while 32.8% were ˂ 10 mIU/mL.

Many studies have investigated the long-term immunogenicity and efficacy of the recombinant HB vaccine in different countries. In agreement of the present study, two Egyptian studies showed seroprotection rates ranged from 39% [2] to 67% [11]. Norouzirad et al [12] reported seroprotection rates 90% among vaccinated Iranian children and 48.9% among adolescents. They observed declining titer of anti-HBsAb levels from 272.3 IU/L to 94.1 IU/L in 1 and 18-year-old population, respectively. Similar to our results, they found a significant negative correlation between age and anti-HBsAb levels [12]. Aghakhani et al [13] reported seroprotection rates 65% in children 1 year after vaccination, and in 30%, 29% and 24% in 5, 10 and 15 years after vaccination, respectively. Other reports from Iran [10, 14-16] reported seroprotection rates from 29% to 75% among vaccinated Iranian children 5 - 10 years after vaccination. In Brazil, Livramento et al [17] reported seroprotection rate (anti-HBs ≥ 10 mIU/mL) in 49.9% of individuals aged 10 - 15 years old. Other Brazilian study showed seroprotection in 58.9% of the studied population [18]. Alfaleh et al [19] reported that 38% of Saudi school students between the ages of 16 and 18 years showed protective anti-HBs titers. Other researchers reported lower levels of seroprotection among vaccinated children; Petersen et al [20] found that only 12.5% and Seto et al [9] found that 19% of the children had protective levels of anti-HBs antibodies. Marked drop in the antibody levels in our study from 89.7% in infants less than 1 year to 64.4% in those more than 1 year and decrease of antibody level over time that was also observed by previous reports [2, 9, 11, 12, 18, 20], from these data we concluded that rapid drop in the seroprotective levels over time recommended a booster dose of HBV.

Contrary to our data many studies from the developed countries reported the absence of breakthrough infection among the vaccinated population 20 years after vaccination. They also indicate a marked decline of acute HBV infection since the introduction of a mass vaccination program for infants. They concluded that there is no need for a booster dose of vaccine until individuals reach the age of 20 years [21-25].

In the present study, patients with diabetes mellitus were found to have the lowest seroprotective levels where 83.3% of diabetic children were non-protected and this was explained by Taheri et al [26], who stated that patients with diabetes mellitus have decreased vaccination response rates compared with healthy controls and this is due to a lower degree in antigen presentation and lower T-cell function. Patients with malnutrition showed non-protective levels in 55.6% whereas 16.7% showed low protective levels needing a booster dose and this may be due to the lower immunoglobulin levels and lower antibody levels in these children. Patients with congenital heart diseases showed non-protective levels in 43.2%. Impaired humeral and cell-mediated immune response in patients with chronic renal failure [21] causing lower sero-convergence rate, low beak of antibody titer and quick decline of antibody levels can explain the results of our study as 26.7% were non-protected and 46.7% show low protective levels and need a booster dose especially that those patients need repeated blood transfusion and more liable to get infection. More than half of patients with chronic liver diseases (57.1%) were non-protected and 28.6% show low immune response. Hypoproteinemia and poor antibody formation in these patients can explain our results. The overall seroprotective levels were markedly lower in all diseased children compared to normal children with significant P value < 0.05 in those with diabetes mellitus, congenital heart disease and malnutrition and this is suspected to be due to the more competent immune system in healthy children.

### Conclusion and recommendation

Our study shows failure to achieve satisfactory seroprotective levels for HB vaccine in both healthy and diseased children who adopted vaccination schedule in Upper Egypt. Booster dose in the second year of life is recommended for all children, particularly for those with diabetes millets, congenital heart disease and malnutrition. Changing the route of administration (subcutaneous injection) or using 1, 2 and 9 months schedule needs to be assessed and compared with the current route and schedule. We need randomized clinical trials to formulate future booster policies for preventing HB infection.

## Abbreviations

Anti-HBs: antibody to hepatitis B surface antigen
ELISA: enzyme-linked immune sorbent assay
HB: hepatitis B
HBsAb: hepatitis B surface antibody
HBsAg: hepatitis B surface antigen
HBV: hepatitis B virus
